# Interaction of Anticancer Drugs with Human Organic Anion Transporter hOAT4

**DOI:** 10.1155/2019/1951786

**Published:** 2019-02-28

**Authors:** Chenchang Liu, Jinghui Zhang, Guofeng You

**Affiliations:** Departments of Pharmaceutics, Ernest Mario School of Pharmacy, Rutgers University, NJ, USA

## Abstract

Human organic anion transporter 4 (hOAT4) belongs to a family of multispecific organic anion transporters that play critical roles in the disposition of numerous drugs and therefore are the major sites for drug-drug interaction. Drug-drug interactions contribute significantly to the individual variation in drug response. hOAT4 is expressed in the kidney and placenta. In the current study, we examined the interaction of 36 anticancer drugs with hOAT4 in kidney COS-7 cells and placenta BeWo cells. Among the drugs tested, only epirubicin hydrochloride and dabrafenib mesylate exhibited > 50% cis-inhibitory effect, in COS-7 cells, on hOAT4-mediated uptake of estrone sulfate, a prototypical substrate for the transporter. The IC_50_ values for epirubicin hydrochloride and dabrafenib mesylate were 5.24±0.95 *μ*M and 8.30±3.30 *μ*M, respectively. Dixon plot analysis revealed that inhibition by epirubicin hydrochloride was noncompetitive with a K_i_ = 3 *μ*M whereas inhibition by dabrafenib mesylate was competitive with a K_i_ = 4.26 *μ*M. Our results established that epirubicin hydrochloride and dabrafenib mesylate are inhibitors of hOAT4. Furthermore, by comparing our data with clinically relevant exposures of these drugs, we conclude that although the tendency for dabrafenib mesylate to cause drug-drug interaction through hOAT4 is insignificant in the kidney, the propensity for epirubicin hydrochloride to cause drug-drug interaction is high.

## 1. Introduction

Human organic anion transporter 4 (hOAT4) belongs to a family of organic anion transporters that significantly contribute to the body disposition, clinical outcome, and toxicity risks of drugs [1–3]. Two major organs, where hOAT4 is richly expressed, are the kidney and placenta [1, 4, 5]. In the kidney, hOAT4 is expressed at the apical membrane of the proximal tubule cells that mediates the renal secretion of anionic drugs into the tubule lumen and their reabsorption from the primary urine, therefore affecting the clinical pharmacokinetic profiles of these compounds [2, 4]. It has been shown that hOAT4 interacts with the inhibitors of angiotensin converting enzyme, antibiotics, antivirals, antineoplastic agents, and nonsteroidal anti-inflammatory drugs [2, 3, 6, 7]. Since hOAT4 has wide range of substrate recognition, drug-drug interactions (DDI) may occur at the transporter when multiple drugs are coexisting, resulting in altered therapeutic response.

The placenta is a specific organ that works as a structural interface between the developing embryo and the parental tissue and therefore is vital for the normal development of fetus and a successful pregnancy [5, 8]. In addition to facilitating the maternal-fetal transfer of nutrients, the placenta also mediates the removal of metabolic wastes, therapeutic agents, and environmental toxins from the fetus [7]. These protective features are in part due to the expression of transporters in the placenta epithelium [9–11]. hOAT4, situated at the fetus-facing basolateral membrane of the placenta, has been proposed to play a role in the cellular uptake and disposition of steroid sulfates, xenobiotics, and clinically important drugs [5, 10, 11]. As women in developed nations continue to delay child birth to a later age, it is likely that the pregnancy and cancer will become a more common coexistence [12, 13]. Thus, more and more women will be exposed to chemotherapy during pregnancy. Given the importance of hOAT4 in fetal development, understanding the tendency of anticancer drugs to interact with hOAT4 is of profound clinical significance.

We previously investigated the interaction of hOAT4 with 101 anticancer drugs from an FDA-approved anticancer drug library. In the current study, we examined the interaction of hOAT4 with additional 36 FDA-approved anticancer drugs in the same library which were updated after our previous publication.

## 2. Materials and Methods

### 2.1. Reagents

The National Institute of Health/National Cancer Institute (NIH/NCI) oncology drug set IV plate, plate key: 4762074 (AOD4-AOD8), was acquired from NCI Chemotherapeutic Agents Repository, Fisher BioServices. AOD 4 (drug set IV plate) contains 101 drugs which were previously examined by our lab [14]. AOD 4 (drug set IV plate) was updated to AOD 8 (drug set IV plate) after our previous publication by adding 36 new drugs into the library. The updated 36 drugs in AOD 8 were examined in the current studies. [^3^H]-estrone sulfate (ES) was obtained from PerkinElmer Life and Analytical Sciences (Waltham, MA). COS-7 cells were purchased from American Type Culture Collection (Manassas, VA). All other reagents were purchased from Sigma-Aldrich (St. Louis, MO) unless stated otherwise.

### 2.2. Cell Culture

Parental COS-7 cells were grown in Dulbecco's modified Eagle's medium (DMEM) containing 10% fetal bovine serum (FBS) in 5% CO_2_ atmosphere at 37°C. COS-7 cells and BeWo cells stably expressing hOAT4 were previously established in our lab and the culture conditions for these cells were previously described by our lab [7, 15].

### 2.3. Transport Measurements

Cells were plated at a density of 120,000 cells/well in 48-well plate. Uptake solution was consisted of phosphate-buffered saline (PBS) (1mM CaCl_2_, 1 mM MgCl_2_, pH7.4) and 300 nM [^3^H]-ES. The uptake experiments were conducted for 4 minutes at room temperature with indicated concentrations of test compounds in the figure legends. Uptake was terminated with rapid washing of the cells with 500 *μ*L ice-cold PBS solution twice. Cells were lysed in 0.2 N NaOH, neutralized in 0.2 N HCl, and placed in individual scintillation vials. Radioactivity was measured using Beckman LC6500 scintillation counter.

### 2.4. Concentration-Dependent Inhibition Studies

Inhibition studies were performed at varying concentrations of epirubicin hydrochloride or dabrafenib mesylate. hOAT4-specific uptake was obtained by subtracting [^3^H]-ES uptake into parental cells from the uptake into hOAT4-expressing cells. The IC_50_ (concentration of the drugs required to inhibit 50% of ES uptake) was determined by nonlinear regression using GraphPad Prism.

### 2.5. Dixon Plot

The mechanism of inhibition was determined by linear regression analysis of reciprocal saturable uptake (1/v) for different substrate concentrations (1.2 *μ*M or 2.4 *μ*M ES) as a function of inhibitor concentration. hOAT4 uptake was determined at 4 minutes in both the absence and presence of varying concentrations of epirubicin hydrochloride or dabrafenib mesylate. The specific uptake was obtained by subtracting [^3^H]-ES uptake into parental cells from the uptake into hOAT4-expressing cells. The data were analyzed by linear regression with GraphPad Prism. K_i_ values were calculated from the intersection of lines representing [ES] =1.2 *μ*M and [ES] = 2.4 *μ*M.

### 2.6. Trans Effect Study

For trans effect study, cells expressing hOAT4 were preloaded with dabrafenib mesylate (100 mM) or ES (100 mM), respectively, for 1 hour at 37°C to allow the chemical substances to diffuse into the cells, followed by rapid washing and subsequent exposure to uptake solution consisted of phosphate-buffered saline (PBS) (1mM CaCl_2_, 1 mM MgCl_2_, pH7.4) and 300 nM [^3^H]-ES. Uptake experiment was preceded as described above.

### 2.7. Statistical Analysis

Each experiment was repeated three times. Statistical analysis was performed using GraphPad Prism software (GraphPad Software Inc., San Diego, CA), one-way ANOVA, multiple comparisons Tukey's test. A *p* value of <0.05 was considered significant.

## 3. Results

### 3.1. Cis Effects of Anticancer Drugs on hOAT4-Mediated Uptake of Estrone Sulfate (ES) in Monkey Kidney COS-7 Cells

**Figure 1:**
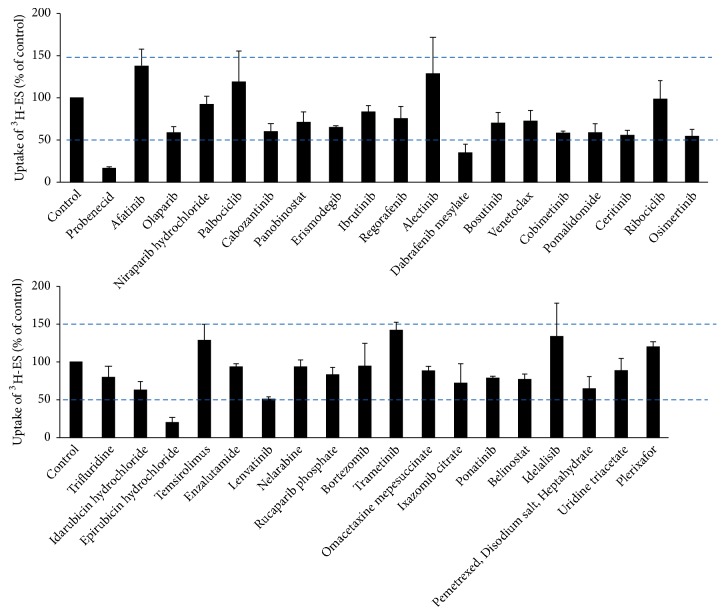
Interaction of hOAT4 with 36 anticancer drugs. hOAT4-mediated [^3^H]-ES uptake was measured in COS-7 cells stably expressing hOAT4. The 4-min uptake of 300 nM [^3^H]-ES in the absence (control) or presence of test compounds (10 *μ*M) was measured. Each data point represents only carrier-mediated transport after subtraction of values from parental cells. Uptake activity was expressed as percentage of uptake measured in control cells. Results shown are means ± SE (n=3).

To investigate the effect of 36 FDA-approved anticancer drugs on hOAT4-mediated uptake of ES, cis-inhibition studies were performed in hOAT4-expressing COS-7 cells. “cis” indicates that both ES and drugs are present on the same side of the cell membrane. Although many of the drugs tested demonstrated some level of inhibition or stimulation, only epirubicin hydrochloride and dabrafenib mesylate demonstrated greater than 50% suppression of hOAT4-mediated [^3^H]-ES uptake at the indicated concentration (Figure 1). Drugs short of significant effects (either inhibitory or stimulatory) suggest a lack of hOAT4 interaction. Thus, their probabilities to cause drug interactions via hOAT4 inhibition can be excluded. Probenecid, a known inhibitor for OAT family members [16], was used as an inhibitor control for this study. We therefore, focus on epirubicin hydrochloride and dabrafenib mesylate in the following studies.

### 3.2. Cis Effects of Epirubicin Hydrochloride and Dabrafenib Mesylate on hOAT4-Mediated ES Uptake in Human Placental BeWo Cells

**Figure 2:**
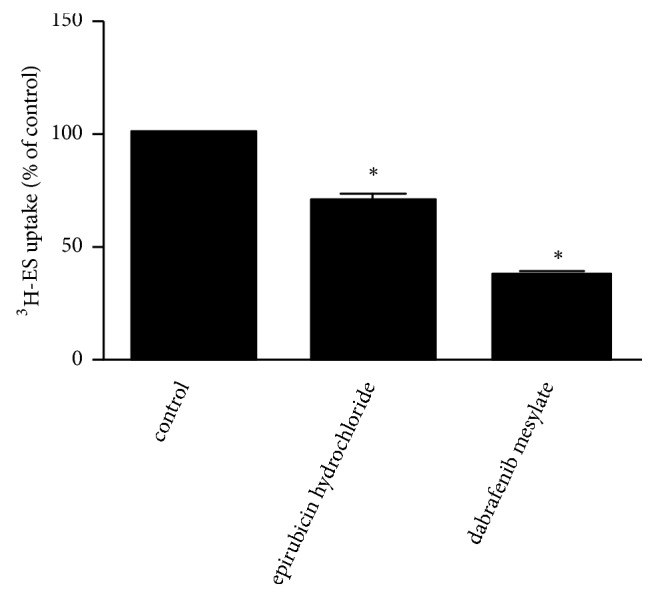
Cis effect of epirubicin hydrochloride and dabrafenib mesylate on hOAT4-mediated [^3^H]-ES uptake in BeWo cells. hOAT4-mediated [^3^H]-ES uptake was measured in stable hOAT4-expressing BeWo cells. The 4-min uptake of 300 nM [^3^H]-ES in the absence (control) or presence of test compounds (100 *μ*M) was measured. Each data point represents only carrier-mediated transport after subtraction of values from parental cells. Uptake activity was expressed as percentage of uptake measured in control cells. Results shown are means ± SE (n=3). Data were analyzed statistically with ANOVA, followed by Tukey's post hoc test. *∗p *< 0.05.

hOAT4 is expressed in both the kidney and placenta. The inhibition effects of epirubicin hydrochloride and dabrafenib mesylate were next characterized in human placental BeWo cells stably expressing hOAT4. At the concentration of 100 *μ*M, both epirubicin hydrochloride and dabrafenib mesylate resulted in significant inhibition of hOAT4-mediated ES uptake in these cells (Figure 2).

### 3.3. Dose-Dependent Effects of Epirubicin Hydrochloride and Dabrafenib Mesylate on hOAT4-Mediated ES Uptake

**Figure 3:**
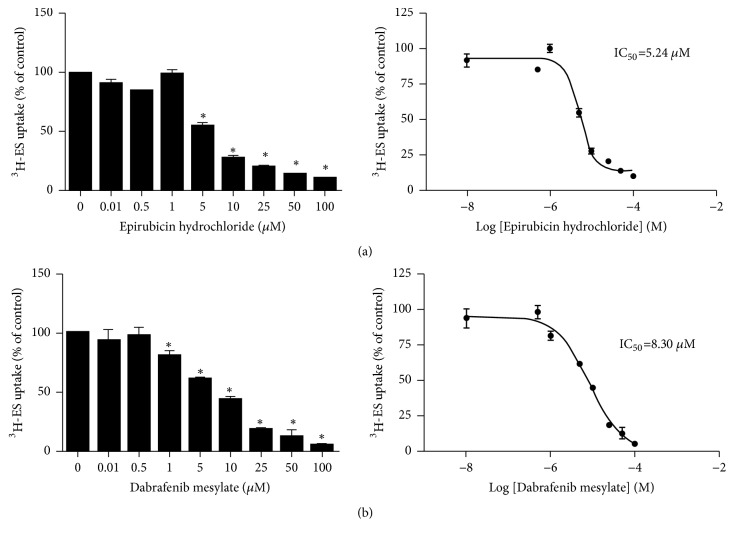
Dose-dependent inhibition of hOAT4-mediated uptake by epirubicin hydrochloride and dabrafenib mesylate. Stable hOAT4-expressing COS-7 cells were incubated for 4 mins with PBS containing 300 nM [^3^H]-ES in the presence or absence of various concentrations of epirubicin hydrochloride (a) or dabrafenib mesylate (b). Each data point represents only carrier-mediated transport after subtraction of values from parental cells. Uptake activity was expressed as percentage of uptake measured in control cells. Results shown are means ± SE (n=3). Data were analyzed statistically with ANOVA, followed by Tukey's post hoc test. *∗p *< 0.05. The line represents a best fit of data using nonlinear regression analysis.

We next constructed dose response curves to evaluate the effectiveness of epirubicin hydrochloride and dabrafenib mesylate as inhibitors of hOAT4-mediated transport in COS-7 cells. Epirubicin hydrochloride (Figure 3(a)) and dabrafenib mesylate (Figure 3(b)) significantly inhibited hOAT4-mediated ES uptake in a concentration-dependent manner with IC_50_ values of 5.24±0.95 *μ*M and 8.30±3.30 *μ*M, respectively.

### 3.4. Dixon Plot Analysis

**Figure 4:**
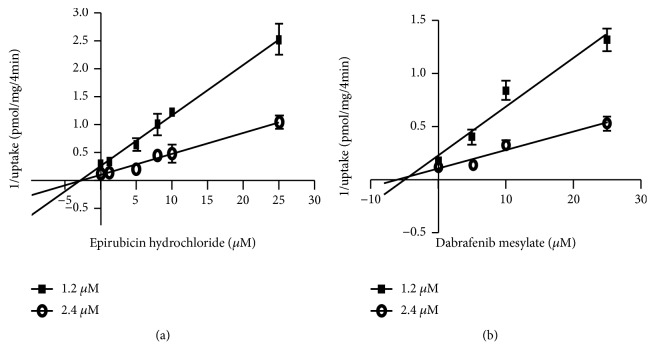
Dixon plot analysis of the inhibitory effects of epirubicin hydrochloride and dabrafenib mesylate on hOAT4-mediated transport in COS-7 cells. 1.2 *μ*M and 2.4 *μ*M [^3^H]-ES uptake was determined at 4 mins in the absence or presence of varying concentrations of epirubicin hydrochloride (a) or dabrafenib mesylate (b). Each data point represents only carrier-mediated transport after subtraction of values from parental cells. Results shown are means ± SE (n=3). The data was fitted by linear regression and K_i_ was calculated. For epirubicin hydrochloride, K_i_= 3 *μ*M and intersection is (-3, 0); for dabrafenib mesylate, K_i_= 4.26 *μ*M and intersection is (-4.26, 0.03).

To further dissect the mechanism of inhibition and to determine the K_i_ values (inhibition constants), uptake in the absence and presence of epirubicin hydrochloride or dabrafenib mesylate was analyzed via Dixon plot (Figure 4). Epirubicin hydrochloride demonstrated a noncompetitive mechanism of inhibition of ES uptake by hOAT4 (as the lines for substrate concentrations converge at the x axis) with a K_i_ value of 3 *μ*M (Figure 4(a)), whereas dabrafenib mesylate demonstrated a competitive mechanism of inhibition of ES uptake by hOAT4 (as the lines for substrate concentrations converge above the x axis) with a K_i_ value of 4.26 *μ*M (Figure 4(b)) [17].

### 3.5. Trans Effect of Dabrafenib Mesylate on hOAT4-Mediated Transport

**Figure 5:**
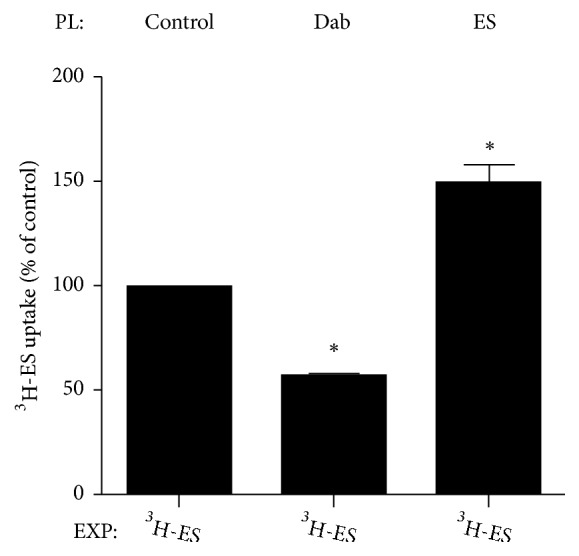
Trans effect of dabrafenib mesylate on hOAT4-mediated transport in COS-7 cells. Cells expressing hOAT4 were preloaded (PL) with dabrafenib mesylate (Dab, 100 mM) or unlabeled hOAT4 substrate estrone sulfate (100 mM) for 1 h, followed by washing with PBS and a subsequent exposure (EXP) to PBS containing ^3^H-labeled estrone sulfate (300 nM). 4 min later, the uptake was stopped by rapidly washing the cells with ice-cold PBS. Intracellular accumulation of ^3^H-labeled estrone sulfate was then counted. Each data point represents only carrier-mediated transport after subtraction of values from parental cells and was expressed as a percentage of the uptake measured in cells without preloading with dabrafenib mesylate or positive control. The results shown are means ± SE (n = 3). *∗p* < 0.05.

Trans effect studies are not needed for epirubicin hydrochloride since it was demonstrated to be a noncompetitive hOAT4 inhibitor in previous experiments. For dabrafenib mesylate, it was shown to be a competitive inhibitor, but it is uncertain whether dabrafenib mesylate could be a substrate and transported by hOAT4. Therefore, trans effect on hOAT4-mediated transport by dabrafenib mesylate was investigated (Figure 5). If the presence of dabrafenib mesylate increases the flux of labeled substrates in the opposite side of the membrane, it would be expected as a substrate of hOAT4; If dabrafenib mesylate tends to bind to the carrier and prevents it from being available for other substrates, instead of transported by hOAT4, then tran-sinhibition would take place. Dabrafenib mesylate showed the trans-inhibition of uptake of radio-labeled OAT substrates rather than tran-stimulation shown by positive control. Therefore, dabrafenib mesylate is not a substrate for hOAT4.

## 4. Discussion

The drug disposition by hOAT4 plays an important role in determining drug efficacy and toxicity. The interaction of hOAT4 with various compounds was reported by other labs including Chinese herbal medicine, angiotensin II receptor antagonists, leukotriene receptor antagonists, nonsteroidal anti-inflammatory drugs and diuretics [5, 10, 18]. Previously we examined the interactions of 101 anticancer drugs (oncology drug set IV plate, AOD 4) with hOAT4 and none of these drugs was identified to have significant potential for hOAT4-mediated drug-drug interaction. Interestingly, in the current study we evaluated the additional 36 anticancer drugs in the same library that was updated after our previous publication and discovered epirubicin hydrochloride, one of the 36 drugs, has high propensity to cause drug-drug interaction in kidney cells.

hOAT4 is mainly expressed in kidney and placenta. Initial screening for hOAT4 inhibitors was carried out in kidney COS-7 cells stably expressing hOAT4. This cell line was previously established in our lab and the characteristics of OATs in these cells have been proven to be similar to those in other systems [15]. Therefore, hOAT4-expressing COS-7 cell line is a suitable model to screen the potential hOAT4 inhibitors. We examined the effects of 36 drugs on hOAT4-mediated [^3^H]-ES uptake. Many of the tested drugs showed different levels of inhibition or stimulation, but only epirubicin hydrochloride and dabrafenib mesylate showed greater than 50% inhibition of hOAT4-mediated [^3^H]-ES uptake (Figure 1). Therefore, we focused on epirubicin hydrochloride and dabrafenib mesylate for further studies. In contrast to the inhibitory effects, several drugs showed stimulation of hOAT4-mediated [^3^H]-ES uptake possibly due to a steric effect [19, 20]. However, since these drugs exhibited less than 50% stimulation, their investigation was not further pursued in this study.

We next characterized the interaction of hOAT4 with epirubicin hydrochloride and dabrafenib mesylate in human placenta BeWo cells, and we observed some differences between placenta BeWo cells and kidney COS-7 cells. In COS-7 cells at 10 *μ*M, both epirubicin hydrochloride and dabrafenib mesylate inhibited uptake of estrone sulfate by more than 50%, with epirubicin hydrochloride being more potent than dabrafenib mesylate. However, in BeWo cells at 100 *μ*M only dabrafenib mesylate inhibited by more than 50% and epirubicin hydrochloride inhibition was only about 30%. Such observation is interesting. Our lab previously demonstrated that the regulation of hOAT4 transport activity is different between kidney cells and BeWo cells due to different sets of regulatory proteins that interact with hOAT4 [21]. Therefore, our current observation that epirubicin hydrochloride and dabrafenib mesylate showed different inhibition potency on hOAT4 transport activity once again confirmed that the functional characteristics of hOAT4 are different between kidney cells and placenta cells. In our current study, The IC_50_ values of epirubicin hydrochloride and dabrafenib mesylate for hOAT4 are determined as 5.24±0.95 *μ*M and 8.30±3.30 *μ*M, respectively (Figures 3(a) and 3(b)). The peak plasma epirubicin hydrochloride concentration (C_max_) suggested by FDA drug product label is 17.2 *μ*M [22]. Corrected by unbound fraction value of 0.23, the unbound maximum plasma concentration (C_u,max_) of epirubicin hydrochloride is around 4 *μ*M. As for dabrafenib mesylate, the maximum plasma concentration (C_max_) is 1.31 *μ*M [23]. Corrected by unbound fraction value of 0.003, according to datasheet provided by FDA [24], the unbound maximum plasma concentration (C_u,max_) of dabrafenib mesylate is around 0.0039 *μ*M. A C_u,max_/IC_50_ value greater than 0.1 suggests a potential for drug-drug interaction [25]. Since C_u,max_/IC_50_ value of epirubicin hydrochloride for hOAT4 is greater than 0.1, while C_u,max_/IC_50_ value of dabrafenib mesylate for hOAT4 is much lower than 0.1, this result suggested that the potential for epirubicin hydrochloride to cause drug-drug interaction through inhibition of hOAT4 is high whereas the potential for dabrafenib mesylate to cause drug-drug interaction through inhibition of hOAT4 is less significant.

**Figure 6:**
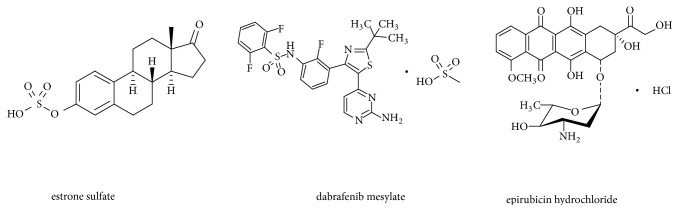
Chemical structures of estrone sulfate, epirubicin hydrochloride, and dabrafenib mesylate.

The inhibition mechanisms for both drugs were also demonstrated by Dixon plot in our study, which revealed that the modes of action of epirubicin hydrochloride and dabrafenib mesylate are distinct. Epirubicin hydrochloride revealed a noncompetitive mechanism of inhibition of ES uptake through hOAT4 (Figure 4(a)), where the activity of the transporter is decreased by the inhibitor by binding to an area other than the substrate binding site. The transporter activity could be reduced through the structure change/steric effect [26]. In contrast, dabrafenib mesylate revealed a competitive mechanism of inhibition of ES uptake through hOAT4 (Figure 4(b)), where the inhibitor binds to the active site on the transporter to prevent the binding between transporter and its substrate. To explore why epirubicin hydrochloride and dabrafenib mesylate showed different inhibitory mechanisms of ES uptake by hOAT4, we compared chemical structures (Figure 6) and physicochemical features of epirubicin hydrochloride, dabrafenib mesylate and estrone sulfate (values are calculated by Chemicalized Platform, ChemAxon, USA) (Table 1). By analysis, dabrafenib mesylate is more similar to estrone sulfate than epirubicin hydrochloride in terms of octanol-water partition coefficient Log P, the number of rings, polar surface area, hydrogen-bond donor count, and hydrogen-bond acceptor count, suggesting that structurally dabrafenib mesylate is more similar to estrone sulfate as compared to epirubicin hydrochloride. Other properties in the table do not show any differences among the three compounds. The structural similarity between dabrafenib mesylate and estrone sulfate explains the competitive inhibition effect of dabrafenib mesylate on ES uptake by hOAT4.

## 5. Conclusion

Our results demonstrated that both epirubicin hydrochloride and dabrafenib mesylate are inhibitors for hOAT4. However, only epirubicin hydrochloride might cause significant drug-drug interaction in kidney cells, whereas the propensity of dabrafenib mesylate to cause drug-drug interaction is very low. Therefore, drug-drug interactions between epirubicin hydrochloride and drugs which are OAT4 substrates should be carefully considered while taken together.

Indeed, OAT4 plays a key role in vivo. For example, lesinurad, Benzbromarone, and Probenecid inhibit kidney OAT4 to block the uric acid reabsorption [27]. Cigarette smoke condensate has also been reported to have inhibitory effect on OAT4 [28]. Understanding hOAT4-mediated drug-drug interaction and the regulation of hOAT4 is of clinical and pharmacological significance.

## Figures and Tables

**Table 1:** Physiochemical characteristics of estrone sulfate, epirubicin hydrochloride, and dabrafenib mesylate.

	estrone sulfate	epirubicin hydrochloride	dabrafenib mesylate
Molecular weight	350.43	579.99	615.65

logP	3.83	0.9	5.46

Physiological Charge	-1	1	0

Hydrogen Acceptor Count	4	12	6

Hydrogen Donor Count	1	6	2

Polar Surface Area	80.67	206.07	110.86

Rotatable Bond Count	2	5	5

Polarizability	36.64	53.88	49.71

Number of Rings	4	5	4

Values are calculated by Chemicalized Platform, ChemAxon, USA.

## Data Availability

The data used to support the findings of this study are included within the article.
